# Chronic Naltrexone Therapy Is Associated with Improved Cardiac Function in Volume Overloaded Rats

**DOI:** 10.1007/s10557-020-07132-4

**Published:** 2021-01-23

**Authors:** Lukas Dehe, Mohammed Shaqura, Michael Nordine, Helmut Habazettl, Petra von Kwiatkowski, Helena Schluchter, Mehdi Shakibaei, Shaaban A. Mousa, Michael Schäfer, Sascha Treskatsch

**Affiliations:** 1 Charité–Universitätsmedizin Berlin, Corporate Member of Freie Universität Berlin, Humboldt-Universität zu Berlin, and Berlin Institute of Health, Department of Anesthesiology and Operative Intensive Care Medicine, Charité Campus Benjamin Franklin, Hindenburgdamm 30, 12203 Berlin, Germany; 2 Charité–Universitätsmedizin Berlin, Corporate Member of Freie Universität Berlin, Humboldt-Universität zu Berlin, and Berlin Institute of Health, Institute of Physiology Campus Charité Mitte, Chariteplatz 1, 10117 Berlin, Germany; 3 Institute of Anatomy, Ludwig-Maximilians-Universität München, Pettenkoferstraße 11, 80336 Munich, Germany

**Keywords:** Cardiac dysfunction, Volume overload, Contractility, Hemodynamics, Opioids, Neurohumoral, Rats

## Abstract

**Purpose:**

Myocardial opioid receptors were demonstrated in animals and humans and seem to colocalize with membranous and sarcolemmal calcium channels of the excitation–contraction coupling in the left ventricle (LV). Therefore, this study investigated whether blockade of the cardiac opioid system by naltrexone would affect cardiac function and neurohumoral parameters in Wistar rats with volume overload-induced heart failure.

**Methods:**

Volume overload in Wistar rats was induced by an aortocaval fistula (ACF). Left ventricular cardiac opioid receptors were identified by immunohistochemistry and their messenger ribonucleic acid (mRNA) as well as their endogenous ligand mRNA quantified by real-time polymerase chain reaction (RT-PCR). Following continuous delivery of either the opioid receptor antagonist naltrexone or vehicle via minipumps (*n* = 5 rats each), hemodynamic and humoral parameters were assessed 28 days after ACF induction. Sham-operated animals served as controls.

**Results:**

In ACF rats mu-, delta-, and kappa-opioid receptors colocalized with voltage-gated L-type Ca2+ channels in left ventricular cardiomyocytes. Chronic naltrexone treatment of ACF rats reduced central venous pressure (CVP) and left ventricular end-diastolic pressure (LVEDP), and improved systolic and diastolic left ventricular functions. Concomitantly, rat brain natriuretic peptide (rBNP-45) and angiotensin-2 plasma concentrations which were elevated during ACF were significantly diminished following naltrexone treatment. In parallel, chronic naltrexone significantly reduced mu-, delta-, and kappa-opioid receptor mRNA, while it increased the endogenous opioid peptide mRNA compared to controls.

**Conclusion:**

Opioid receptor blockade by naltrexone leads to improved LV function and decreases in rBNP-45 and angiotensin-2 plasma levels. In parallel, naltrexone resulted in opioid receptor mRNA downregulation and an elevated intrinsic tone of endogenous opioid peptides possibly reflecting a potentially cardiodepressant effect of the cardiac opioid system during volume overload.

## Introduction

With an increasing life expectancy throughout the world, patients undergoing surgery will be inherently older and exhibit a greater prevalence of (congestive) heart failure (CHF). This increased prevalence of heart failure (HF) may give rise to significant increases in perioperative complications and mortality [1, 2]. Conclusions drawn from previous findings showed that opioids elicit cardioprotective effects against myocardial ischemic events in vitro (*conditioning*) [3]. As such, a high-dose opioid-based anesthesia technique has been advocated to reduce adverse cardiac events during anesthesia [4].

Recent experimental studies have detected all three opioid receptors in normal rat and human cardiac tissue [5–8]. Besides a colocalization with sympathetic, parasympathetic, and sensory neurons, opioid receptors seem to be coexpressed with important structures of the myocardial excitation–contraction coupling and mitochondria in the left ventricle [5–7, 9, 10]. Moreover, an activation of this intrinsic cardiac opioid system in volume-overloaded rat hearts was detected, reflected by an upregulation of delta- and kappa-opioid receptor expression and their respective endogenous ligand peptide precursors [5, 7]. In this context, acute infusion of kappa-opioid receptor agonists acutely provoked an augmented negative inotropic and lusitropic response in the failing ex vivo perfused hamster heart [11]. In addition, short-term systemic delta-opioid receptor inhibition increased cardiac output and improved left ventricular performance in dogs with CHF [12].

The question now arises whether the intrinsic cardiac opioid system may possess direct cardiodepressant effects. This proof-of-concept study is aimed at investigating whether the cardiac opioid system may influence cardiac function and neurohumoral parameters in volume-overloaded rat hearts as one experimental model of heart failure. We hypothesized that the persistent inhibition of the endogenous opioid tone by chronic treatment with the opioid receptor antagonist naltrexone in rats with aortocaval fistula (ACF)-induced volume overload will improve cardiac contractility and attenuate neurohumoral activation.

## Materials and Methods

### Animals

Experiments were conducted in male Wistar rats (280–300 g) (Harlan Winkelmann, Borchen, Germany) following approval by the local animal care committee (Landesamt für Gesundheit und Soziales, Berlin, Germany; G0144/12) and were performed according to the European Directive introducing new animal welfare and care guidelines (2010/63/EU). Rats were maintained on standard laboratory rat chow and water ad libitum and kept on a 12-h/12-h light–dark cycle.

### ACF Induction and Naltrexone Treatment

Chronic volume overload was induced (*n* = 10 rats) using a modified approach of an infrarenal aortocaval fistula (ACF) as previously described [13]. Briefly, following an abdominal laparotomy, the abdominal aorta was punctured with a 16G disposable needle (Braun, Melsungen, Germany) and then pushed forward into the adjacent vena cava inferior. After withdrawal of the needle, the aortic puncture site was sealed with cyanoacrylate glue and the ACF patency was judged by the pulsatile flow of oxygenated blood from the aorta into the vena cava inferior [14]. Immediately at the end of ACF induction, 5 rats of the aforementioned group received a subcutaneous administration of the opioid receptor antagonist naltrexone (10 mg × kg^−1^ ×h^−1^) for 28 days, which was continuously applied by subcutaneously implanted Alzet® minipumps (osmotic pump, model 2ML4, 2.5 μl per hour) (*ACF/naltrexone*). The dose chosen for the naltrexone treatment via minipumps was based on previously published protocols in rats [15]. Naltrexone, compared with naloxone, exhibits a longer-acting opioid receptor antagonist effect, with a half-life of 3.9–10.3 h vs. approximately 60 min [16, 17]. The other rats (*n* = 5) of the ACF group also received a minipump delivering vehicle (isotonic saline) at the same volume during the whole experiment (*ACF/vehicle*). Sham-operated animals were treated identically except for the puncture of the aorta and without any minipump treatment serving as controls (*n* = 5) (*control*). For post-surgical analgesia, metamizole (40 mg/kg) was subcutaneously injected [13].

### Hemodynamic Evaluation

The “closed chest” method was used for hemodynamic evaluation as described previously [13]. Twenty-eight days after fistula induction in spontaneously breathing rats, the measurements were performed under tiletamine/zolazepam anesthesia (Zoletil®, 10 mg/kg s.c. followed by 50 mg/kg i.m.) [18]. Following anesthesia induction (5–10 min), rats received a tracheostomy to facilitate spontaneous breathing and were placed on a heating pad to maintain body temperature. Central venous pressure (CVP) was assessed with a plastic catheter (PE-50) that was inserted via the left jugular vein into the superior vena cava. Arterial and intraventricular pressures and their deriatives were measured with a pressure micro-tip catheter (Millar®, SPR-838 NR), which was advanced into the left ventricle via the right carotid artery. All catherizations were performed by the same trained physician and lasted approx. 20–30 min. All data were recorded and analyzed by the PowerLab®-system and software (AD Instruments, Dunedin, New Zealand). For all experiments, triplicate samples were obtained at 10-min intervals to reach stable measurement conditions. The triplicate results were averaged, and the averages were used for subsequent analyses. After completion of hemodynamic measurements, rats were killed by exsanguination and organs were eviscerated.

### Determination of BNP and Angiotensin-2 Plasma Concentration

Measurement of rat brain natriuretic peptide 45 (rBNP-45) and angiotensin-2 concentration was performed as previously described [13]. Both parameters were chosen in concordance with the diagnostic pathway suggested by the recent clinical heart failure guideline [1] and the well-established neurohumoral activation of the renin-angiotensin-aldosteron system (RAAS) [19]. Blood samples were withdrawn from all animals (*n* = 5 rats per group) into EDTA-preloaded tubes after completion of hemodynamic measurements. Immediately after withdrawal, the blood was centrifuged at 1000 g for 10 min at 4 °C. Subsequently, the plasma was maintained at − 80 °C until further use. For the measurement of triplicate samples of plasma rBNP-45 or angiotensin-2 concentrations, a sensitive enzyme-linked immunosorbent assay (ELISA) kit (Abnova, Heidelberg, Germany) was used [20].

### Opioid Receptor Identification in Left Ventricular Myocardium

To obtain a left ventricular myocardial tissue for double immunofluorescence from rats with ACF-induced volume overload, animals (*n* = 3 additional rats per group) were deeply anesthetized with tiletamine/zolazepam (Zoletil®) and transcardially perfused with 100 ml warm saline, followed by 300 ml 4% (*w*/*v*) paraformaldehyde in 0.16 M phosphate buffer solution (pH 7.4) (“fixative solution”). Then, hearts were removed, postfixed in fixative solution, and cryoprotected overnight at 4 °C in PBS containing 10% sucrose. The left ventricular myocardial tissue was then embedded in tissue-Tek compound (OCT, Miles Inc. Elkhart, IN), frozen and cut into 10-μm thick sections using a cryostat. The sections were mounted onto gelatin-coated slides and incubated overnight with the following primary antibodies: rabbit polyclonal anti-Mu-Opioid Receptor (MOR) (1 : 1000) (gift from S. Schulz and V. Höllt, Magdeburg, Germany) [10], rabbit polyclonal anti-Delta-Opioid Receptor (DOR) (Dr. R. Elde, Minneapolis, MN, USA),and rabbit polyclonal anti-Kappa-Opioid Receptor (KOR) (1 : 1000) (gift from S.J. Watson, Michigan, USA) [21] in combination with the mouse monoclonal anti-dihydropyridine receptor (ɑ2 subunit) antibody to identify the voltage-gated L-type Ca2+ channel (anti-Cav1.2) (SIGMA®, Missouri, USA) [22, 23]. After incubation, with primary antibodies, the tissue sections were then washed with PBS and incubated with Texas Red-conjugated goat anti-rabbit antibody (Vector Laboratories) and FITC-conjugated donkey anti-mouse secondary antibodies (Vector Laboratories, Inc. Burlingame, CA). Thereafter, sections were washed with PBS and the nuclei stained bright blue with 4′-6-diamidino-2-phenylindole (DAPI) (0.1 μg/ml in PBS) (SIGMA®, Missouri, USA). Finally, the tissues were washed in PBS, mounted on vectashield (Vector Laboratories), and viewed under a Zeiss LSM 510 laser scanning microscope (Carl Zeiss, Göttingen, Germany). To demonstrate specificity of staining, the following controls were included: omission of the primary antisera or the secondary antibodies, as described in previous studies [5–7].

### Opioid Receptor and Opioid Peptide Precursor Expression

Total RNA was extracted from left cardiac ventricles (*n* = 4 rats per group) by using the commercially available Qiazol Lysis kit, (Qiagen, Hilden, Germany) as previously described [5, 7]. The following specific primers were generated and used: for MOR, forward primer: TTACGGCCTGATGATCTTACGA, reverse primer: GGTGAT CCTGCGCAGATTC (Ensembl, Accession Nr: NM_001304737); for DOR, forward primer: GCTGGGCTACGCCAACAG, reverse primer: CGGAAGCAGCGCTTGAAG (Ensembl, Accession Nr: NM_012617); for KOR, forward primer: TCTTTATCCTGGTCGAGGCTCTA, reverse primer: CCCAAGGCAATGCAG AAGTAA (Ensembl, Accession Nr: NM_017167); for pro-opiomelanocortin (POMC), forward primer: AGAGTTCAAGAGGGAGCTGGAA, reverse primer: GTCGGCCTTCTCGGTATCC (Ensembl, Accession NM_139326); for pro-enkephalin (PENK), forward primer: TCCGACCTGCTGAAAGAGCTA, reverse primer; TGCTTTCCTGTTGGTGGCTAT (Ensembl, Accession Nr: NM_017139); for pro-dynorphin (PDYN), forward primer: AAGCTTAAGT GGGACAACCAGAAA, reverse primer: GTTCTCCTGGGACCGAGTCA (Ensembl, Accession Nr: NM_019374); and for 18S, forward primer: CGGCTACCACATCCAAGGAA, reverse primer: GCTGGAATTACCGCGGCT (Ensembl, Accession Nr: NR_046237). Quantitative real-time-PCR (RT-PCR) was performed with a SYBR® Green kit following the manufacturer’s instructions (Applied Biosystems, Carlsbad, CA). Amplification was carried out for 40 cycles, each consisting of 15 s at 95 °C. A temperature just below the specific melting temperature (Tm) was employed for detection of fluorescence-specific products. MOR, DOR, and KOR as well as POMC, PENK, and PDYN mRNAs were quantified in triplicates of samples using the delta-delta CT method [24].

### Statistical Analyses

Results are expressed as medians plus their interquartile ranges. Statistical data analyses for testing potential differences between the three groups were performed by using a one-way analysis of variance (ANOVA) on Ranks (Kruskal–Wallis test) and post hoc by a Student–Newman–Keul test. Statistical tests were performed using Sigma Plot 13.0 statistical software (Systat Software GmbH, Erkrath, Germany).

## Results

### MOR, DOR, and KOR Localization in the Left Ventricle Myocardium

**Figure 1:**
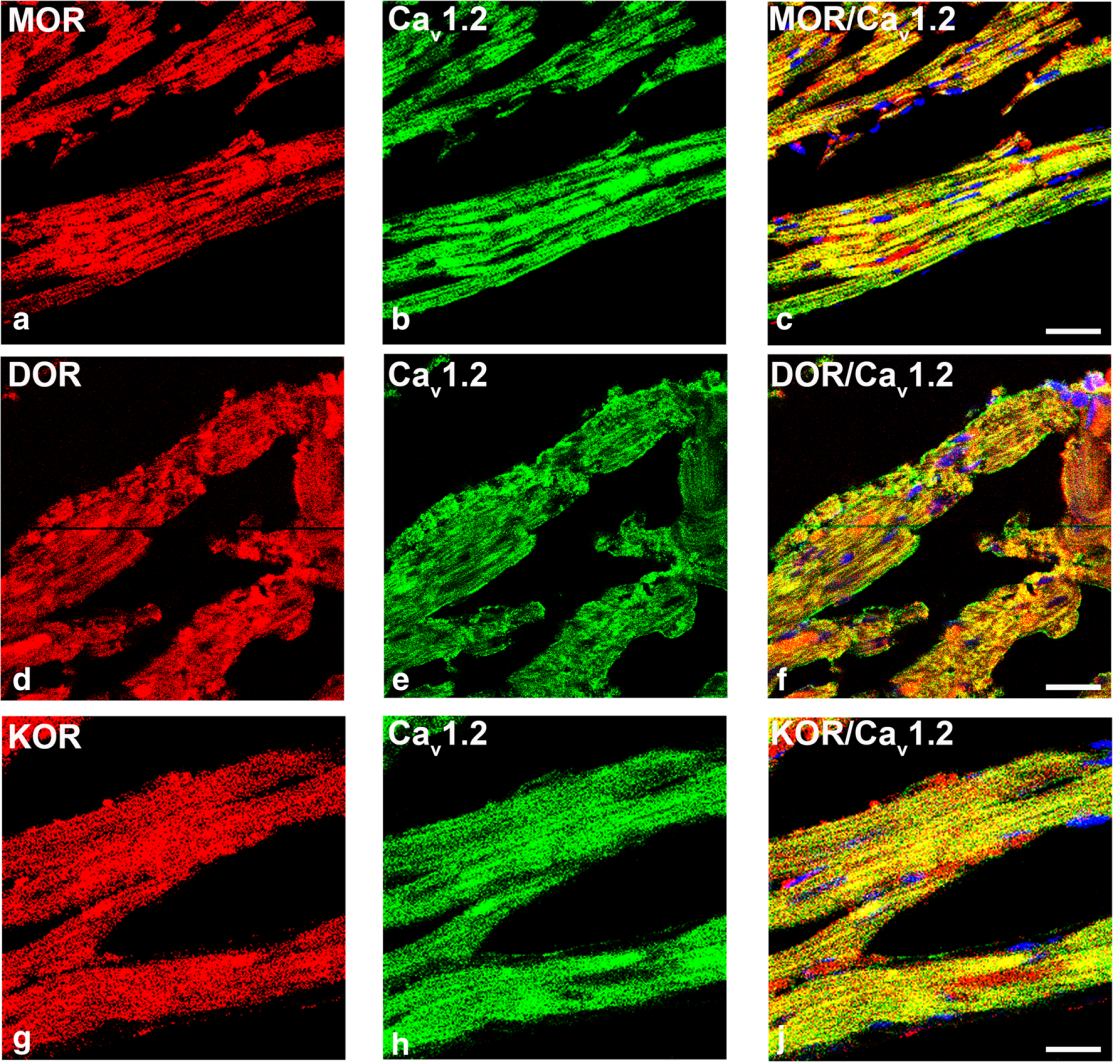
Confocal fluorescence microscopic images of left ventricular myocardium of rats with ACF-induced volume overload stained with rabbit polyclonal anti-MOR (**a**), anti-DOR (**d**), or anti-KOR (**g**) (*Texas red immunofluorescence*) in combination with mouse monoclonal anti-voltage-gated L-type Ca^2+^-channel Cav1.2 (*FITC green fluorescence*) (**b**, **e**, **h**). Nuclei are recognized by their bright blue (DAPI) fluorescence. MOR, DOR, and KOR immunoreactivity is restricted to the myocard of the left ventricle, revealing an abundant colocalization with the L-type Ca^2+^-channel Cav1.2 (seen as yellow fluorescence)(**c**, **f**, **j**). Bar = 20 μm (500x magnification)

Double immunofluorescence demonstrated that MOR, DOR, and KOR were abundantly expressed in the left ventricular myocardium of rats with ACF-induced volume overload (Fig. 1a, d, and g). Moreover, they highly colocalized with the voltage-gated L-type Ca^2+^-channel Cav1.2 of the ventricular myocardium (Fig. 1c, f, and j).

### Cardiac Remodeling in ACF Rats

Values for organ weight/body weight ratio (‘index’) are given in Table 1. Heart (*P* = 0.016) and lung (*P* = 0.02) weight indices were significantly increased in all ACF rats 28 days after fistula induction. Heart and lung weight indices, however, did not differ between ACF/vehicle and ACF/naltrexone (*P* = 0.347 and *P* = 0.917, respectively). Kidney and liver weights were not significantly different between groups and/or treatment (Table 1).

**Table 1:** Morphometric data of the body weight; heart, lung, kidney, and liver organ weights; and organ weights in relation to body weights of control rats and vehicle- (isotonic saline) and naltrexone-treated rats with ACF-induced volume overload**.** Values for the body weight, heart weight, heart/BW, lung weight, lung/BW, kidney weight, kidney/BW liver weight, and liver/BW are given as medians plus interquartile ranges. *n* = 5 rats/group. *BW* body weight. Potentially significant differences between the ACF/Vehicle and control group (*P*_1_ value) and between the ACF/vehicle and ACF/naltrexone groups (*P*_2_ value) are presented. *P* < 0.05 was considered statistically significant

	Control	ACF/vehicle	ACF/naltrexone	*P* value
Body weight (g)	405.0 (330.5, 447.0)	435.0 (410.5, 464.5)	415.0 (374.5, 441.5)	*P* = 0.424
Heart weight (mg)	1420 (1315, 1720)	2250 (1955, 2595)	1920 (1760, 2210)	*P*_1_ = 0.014 *P*_2_ = 0.230
Heart/BW (mg/g KG)	3.9 (3.4, 4.2)	5.1 (4.6, 5.8)	4.6 (4.4, 5.4)	*P*_1_ = 0.016 *P*_2_ = 0.347
Lung weight (mg)	1490 (1280, 1900)	2380 (2220, 2525)	2260 (2050, 2665)	*P*_1_ = 0.016 *P*_2_ = 0.754
Lung/BW (mg/g KG)	4.2 (3.5, 4.4)	5.1 (5.0, 6.0)	5.2 (5.0, 6.0)	*P*_1_ = 0.020 *P*_2_ = 0.917
Kidney weight (mg)	1320 (1110, 1700)	1290 (1225, 1445)	1290 (1225, 1505)	*P* = 0.983
Kidney/BW (mg/g KG)	3.2 (3.2, 3.9)	3.0 (2.9, 3.2)	3.3 (3.0, 3.5)	*P* = 0.075
Liver weight (mg)	13,330 (11,520, 14,210)	12,320 (11,685, 13,260)	12,140 (11,070, 12,990)	*P* = 0.566
Liver/BW (mg/g KG)	33 (29, 38)	28 (28, 29)	32 (27, 32)	*P* = 0.164

### Naltrexone Improved Cardiac Function

**Fig. 2:**
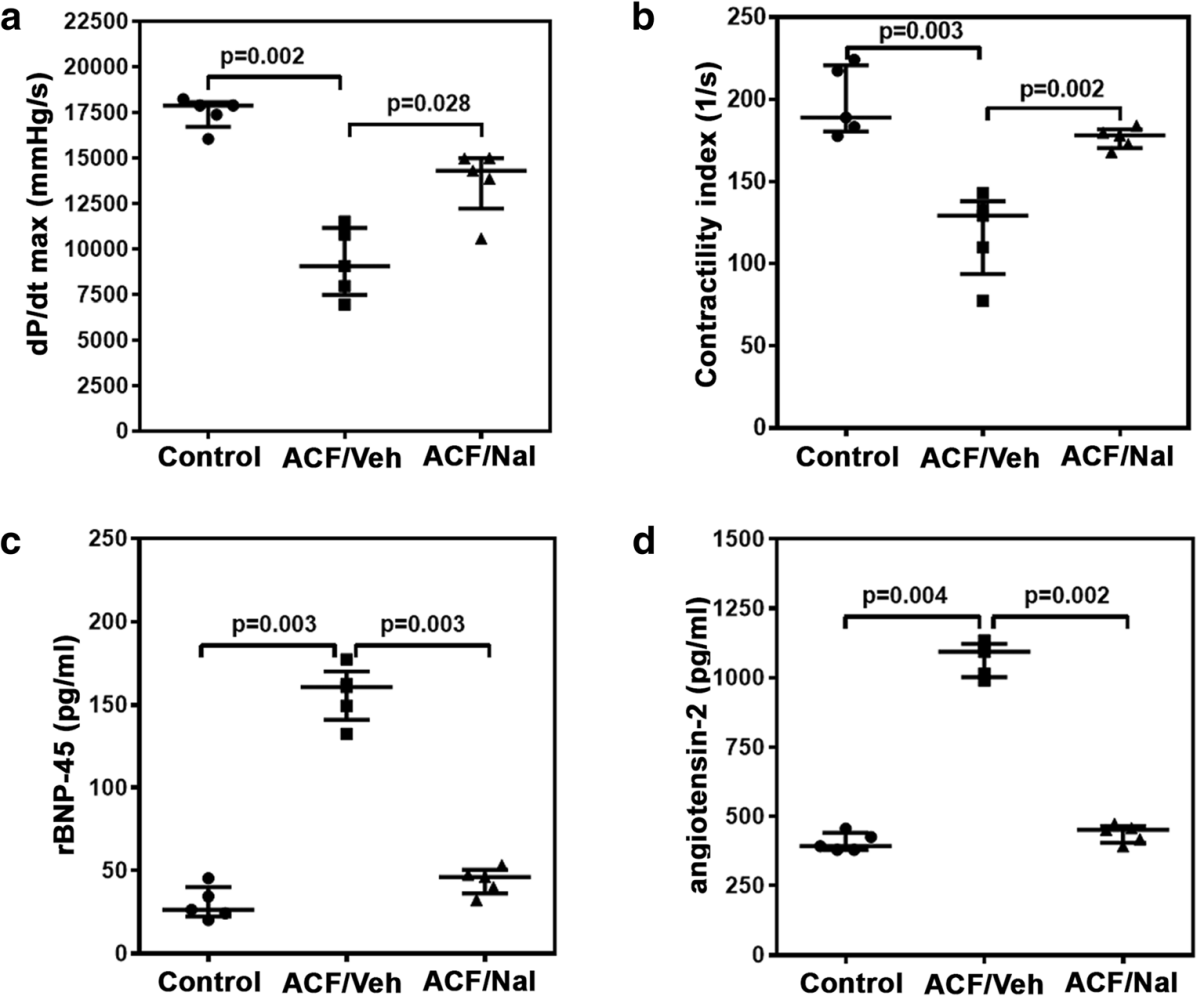
dP/dt max (**a**) and contractility index values (**b**) of the control and ACF/vehicle- and ACF/naltrexone-treated rats. Values of both hemodynamic parameters were significantly reduced in the ACF/vehicle-treated rats compared to controls (*P* = 0.002 and *P* = 0.003, respectively). Following chronic naltrexone treatment, the reduction in both hemodynamic parameters of ACF/vehicle-treated rats was significantly prevented in ACF/naltrexone-treated rats (*P* = 0.028 and *P* = 0.002, respectively). Values are medians plus interquartile ranges (*n* = 5 rats/group). rBNP-45 (**c**) and angiotensin-2 (**d**) plasma concentrations of control and ACF/vehicle- and ACF/naltrexone- treated rats. rBNP-45 (*P* = 0.003) and angiotensin-2 (*P* = 0.004) plasma concentrations were significantly increased in ACF/vehicle-treated rats compared to controls. Following chronic naltrexone treatment, the increase in both rBNP-45 (*P* = 0.003) and angiotensin-2 (*P* = 0.002) of ACF/vehicle-treated rats was significantly prevented in ACF/naltrexone-treated rats. Values are medians plus interquartile ranges (*n* = 5 rats/group)

Table 2 shows values for measured hemodynamic parameters amongst the control, ACF/vehicle, and ACF/naltrexone groups. Systolic and diastolic blood pressures (SBP, DPB) were decreased in all ACF rats due to the aortocaval fistula (*P* = 0.001 and *P* < 0.005, respectively). CVP and left ventricular end-diastolic pressure (LVEDP) were significantly increased in ACF/vehicle (CVP: *P* = 0.002; LVEDP: *P* = 0.001). Chronic treatment with naltrexone was associated with a significant decrease in CVP (*P* = 0.005) and LVEDP compared to vehicle treatment in ACF rats (*P* = 0.009). Global systolic left ventricular function as measured by *dP/dt*_max_ (*P* = 0.028) and contractility index (defined as *dP/dt*_max_ divided by the pressure *P* at the time of *dP/dt*_max_) (*P* = 0.002) significantly improved due to naltrexone treatment in rats with an aortocaval fistula (Fig. 2a, b). Naltrexone treatment significantly improved left ventricular diastolic function in ACF rats as measured by *dP/dt*_min_ (*P* = 0.028).

**Table 2:** Hemodynamic data of control rats and vehicle- (isotonic saline) and naltrexone-treated rats with ACF-induced volume overload. Values are given as medians plus interquartile ranges (*n* = 5 rats/group): *HR* heart rate, *SBP* systolic blood pressure, *DBP* diastolic blood pressure, *CVP* central venous pressure, *LVEDP* left ventricular end-diastolic pressure. Potentially significant differences between the ACF/vehicle and control group (*P*_1_ value) and between the ACF/vehicle and ACF/naltrexone group (*P*_2_ value) are presented. *P* < 0.05 was considered statistically significant

	Control	ACF/vehicle	ACF/naltrexone	*P* value
HR (min^-1^)	382 (351, 408)	321 (316, 51)	348 (290, 362)	*P* = 0.065
SBP (mmHg)	163 (148, 172)	118 (113, 126)	142 (126, 167)	*P*_1_ = 0.010 *P*_2_ = 0.009
DBP (mmHg)	134 (108, 146)	78 (76, 82)	92 (78, 104)	*P*_1_ = 0.005 *P*_2_ = 0.097
CVP (mmHg)	0.3 (0.12, 0.75)	4.8 (3.0, 9.5)	0.9 (0.6, 1.8)	*P*_1_ = 0.002 *P*_2_ = 0.005
LVEDP (mmHg)	5.0 [4.8; 5.6]	10.6 [8.3; 11.3]	6.8 [6.4; 8.4]	*P*_1_ = 0.001 *P*_2_ = 0.009
*dP/dt*_max_ (mmH/s)	17,889 (16,729, 18,061)	9068 (7475, 11,157)	14,314 (12,226, 14,995)	*P*_1_ = 0.002 *P*_2_ = 0.028
*dP/dt*_min_ (mmHg/s)	− 10,711 (− 12,027, −8748)	− 6934 (− 7532, − 6226)	− 9307 (− 9721, − 7810)	*P*_1_ = 0.005 *P*_2_ = 0.001
Contractility index (1/s)	189 (181, 221)	129 (94, 138)	178 (170, 182)	*P*_1_ = 0.003 *P*_2_ = 0.002

### Naltrexone Prevented Increased rBNP-45 and Angiotensin-2 Plasma Concentrations in Rats with ACF-Induced Volume Overload

Values for the rBNP-45 and angiotensin-2 plasma concentration from the control, ACF/vehicle, and ACF/naltrexone groups are shown in Fig. 2c, d. Volume overload resulted in a significant increase in rBNP-45 (*P* < 0.003) and angiotensin-2 (*P* < 0.004) plasma levels in ACF/vehicle rats. Continuous subcutaneous delivery of naltrexone significantly prevented this increase in rBNP-45 (*P* < 0.003) and angiotensin-2 (*P* < 0.002) plasma concentrations in ACF rats, and no evidence for a differences to the control group were observed.

### Naltrexone Downregulated Opioid Receptor but Enhanced Opioid Peptide Expresssion in Rats with ACF-Induced Volume Overload

**Fig. 3:**
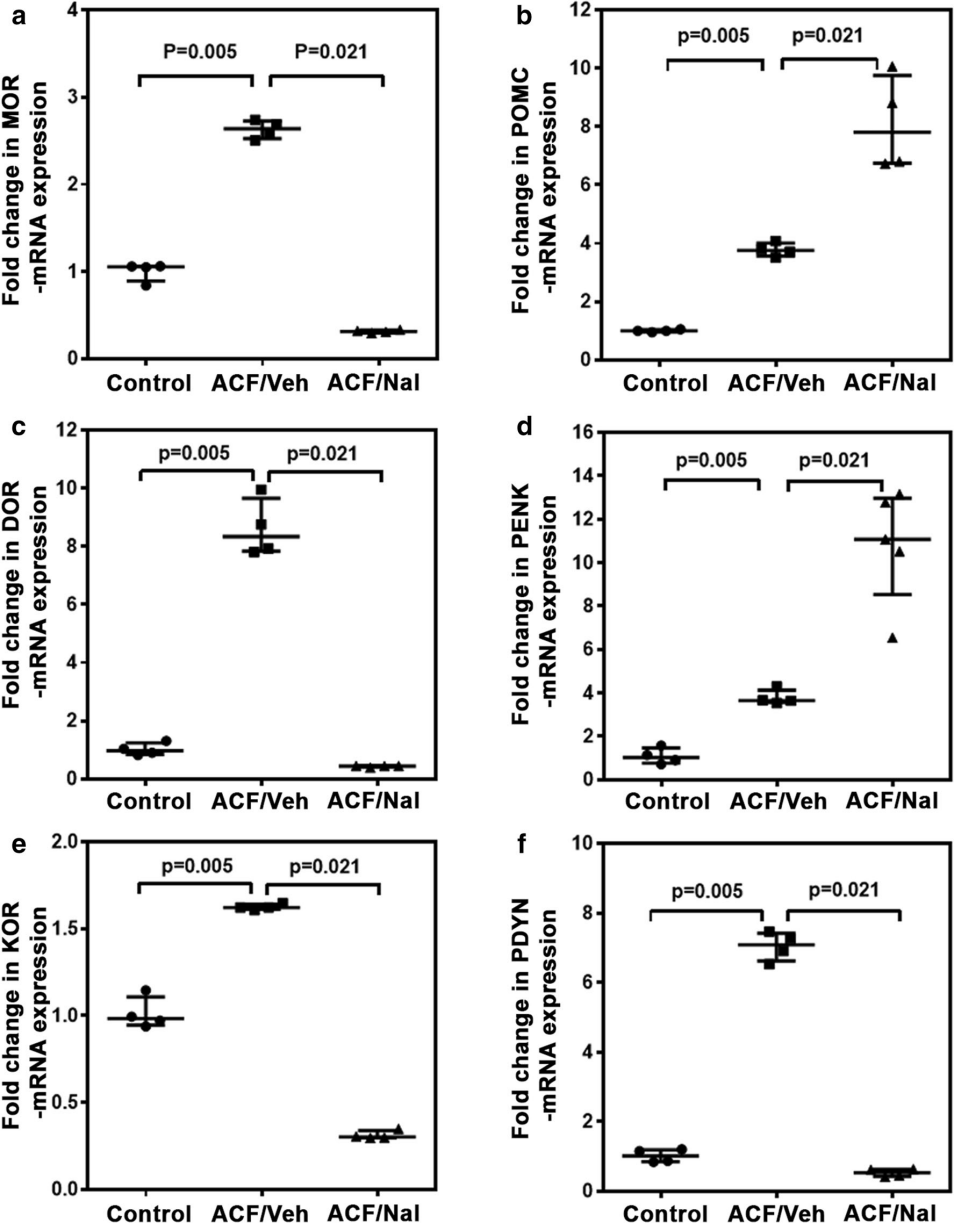
Quantitative RT-PCR of the left ventricular myocardium with MOR- (**a**), DOR- (**c**), and KOR-specific (**e**) primers shows the presence of all three opioid receptors. MOR, DOR, and KOR mRNA transcripts were significantly increased in ACF/vehicle-treated rats compared to controls (*P* = 0.005). Following chronic naltrexone treatment, this increase of ACF/vehicle-treated rats was significantly abolished in ACF/naltrexone-treated rats (*P* = 0.021) (**a, c, e**). **b**, **d**, **f** Expression of the corresponding endogenous opioid peptide precursor mRNA POMC, PENK, and PDYN in the left ventricular myocardium. POMC, PENK, and PDYN mRNA transcripts were upregulated during ACF-induced cardiac volume overload (*P* = 0.005). Following chronic opioid antagonist treatment by naltrexone, this enhanced expression was further increased for POMC and PENK (**b**, **d**) (*P* = 0.021, Kruskal–Wallis test, post hoc Student–Newman–Keuls test) but was decreased for PDYN (*P* = 0.021) (**f**). Values are medians plus interquartile ranges (*n* = 5 rats/group)

Using quantitative RT-PCR for detection of mRNA expression our results show that MOR, DOR, and KOR mRNAs were expressed in the left ventricular myocardium of all three groups (control rats, ACF/vehicle rats, and ACF/naltrexone rats) (Fig. 3a, c, and e). Chronic treatment of ACF rats with the opioid antagonist naltrexone significantly decreased the expression of MOR, DOR, and KOR mRNA compared to ACF rats treated with vehicle (*P* = 0.021, Kruskal–Wallis and post hoc Student–Newman–Keuls test) (Fig. 3a, c, and e). Expression of the corresponding endogenous opioid peptide precursor mRNA POMC, PENK, and PDYN were upregulated during ACF-induced cardiac volume overload (Fig. 3b, d, and f). Following chronic opioid antagonist treatment by naltrexone, this enhanced expression was further increased for POMC and PENK (*P* = 0.021, Kruskal–Wallis and post hoc Student–Newman–Keuls test), but significantly decreased for PDYN (*P* = 0.021, Kruskal–Wallis and post hoc Student–Newman Keuls) (Fig. 3b, d, and f).

## Discussion

This study shows MOR, DOR, and KOR expressions in LV cardiomyocytes of ACF rats colocalizing with the expression of voltage-gated L-type Ca^2+^-channel Cav1.2 as part of the excitation–contraction coupling in the LV. In rats with ACF-induced volume overload, chronic administration of the opioid antagonist naltrexone was associated with an improved LV function as evidenced by decreases in CVP and LVEDP, increases in cardiac contractility, and decreases in rBNP-45 and angiotensin-2 plasma levels. In parallel, chronic naltrexone treatment led to a significant decrease of the ACF-induced increased expression of MOR, DOR, and KOR mRNAs and to a further significant increase in the already elevated mRNA of the endogenous opioid peptide precursors POMC and PENK, but not PDYN. These results possibly reflect a reduction in the cardiodepressive effects of the opioid system due to opioid receptor blockade and a compensatory prevalence of the sympathetic stress on the heart.

Our findings of opioid receptor and peptide mRNA as well as protein in the left ventricular myocardium are in line with several previous publications in rats which have demonstrated MOR, DOR, and KOR by mRNA detection, western blot, immunohistochemistry, and radiolabeled ligand binding [5–7]. They are consistent with studies in humans in which MOR, DOR, and KOR were identified in myocardial tissue that was obtained during autopsy after sudden death [8] and with PET imaging studies in human volunteers at the Johns Hopkins Hospital demonstrating cardiac MOR and DOR [25]. Interestingly, all three opioid receptors (MOR, DOR, and KOR) as well as their respective endogenous ligands (derived from the precursors POMC, PENK, and PDYN) were shown to be upregulated during the stressful conditions of ACF-induced left ventricular volume overload [5–7]. This animal model is characterized by significantly elevated CVP and LVEDP, a shift to higher volumes in the pressure–volume loops without any overlap, a significant decrease in the ejection fraction by almost 40%, and a significantly reduced maximum rate of pressure decay and prolonged time of tau depicting the transition from eccentric hypertrophy with preserved cardiac function to severe biventricular dilatation with decompensated heart failure [13]. This was further corroborated by showing clear biochemical, immunohistochemical, and electron microscopical evidence for extended myocardial apoptosis in this animal model [26]. Following naltrexone treatment of ACF, rats’ myocardial expression of MOR, DOR, and KOR mRNAs was significantly downregulated—even beyond control values—which might be related to the previously demonstrated enhanced opioid peptide levels during volume overload-induced heart failure [5, 7]. On the other side, opioid peptides derived from POMC and PENK targeting predominantly MOR and DOR, while dynorphins derived from PDYN having the highest affinity for KOR appear to be differentially regulated. Naltrexone increased the opioid peptide precursor levels POMC and PENK above those in the ACF/Veh group, while reducing PDYN levels below control values. While in the spinal cord or central nervous system, dynorphin follows an opposite functional role compared to ß-endorphin and met-enkepahlin [27]; nothing has been reported so far for the heart.

Due to their presumed neutral hemodynamic effects, high-dose opioids are widely used to provide analgesia in high-risk patients undergoing anesthesia [28, 29]. Opioids also seem to protect the heart from ischemia/reperfusion (IR) injury, and are, thus, a cornerstone of cardiac and high-risk non-cardiac anesthesia [30]. Interestingly, especially amongst elderly patients (age > 75 years) and patients with concomitant congestive heart failure (CHF); intravenous morphine increased in-hospital mortality when administered in acute coronary syndromes [31]. In addition, patients receiving morphine were more likely to develop CHF. In this context the question of potential opioidergic adverse effects on cardiac function arises.

Recent animal studies provided evidence for an intrinsic cardiac opioid system which seemed to be upregulated—receptor and endogenous ligand—during volume overload [5, 7]. In this context, short-term infusion of the opioid receptor antagonist naloxone increased arterial pressure, cardiac contractile function, and organ blood flow by acting on DOR in conscious dogs with pacing-induced CHF [12]. In line with this, Bolte et al. were able to provoke an augmented negative inotropic and lusitropic response in the failing ex vivo perfused hamster heart by administering selective agonists for KOR and DOR [11]. Opposite results were obtained by a short-term high-dose infusion of non-selective beta-endorphin in patients with mild to moderate CHF which improved LVEF, reduced systemic vascular resistance, and blunted the neurohormonal activation [32].

Extending the aforementioned findings, we demonstrated that chronic administration of naltrexone was able to improve cardiac function in volume-overloaded hearts in anesthetized rats. In previous work, morphine sulfate was shown to decrease heart rate and cardiac output in a dose-related fashion in healthy rat hearts, and naloxone attenuated these negative cardiovascular effects in ex vivo perfused healthy rat hearts [33]. This is somewhat in contrast to a previous observation in conscious dogs with right-sided CHF, in which a 6-week oral administration of naltrexone showed no effects on resting cardiac function [34]. An improvement of LV contractility was only detectable after beta-adrenergic isoprenaline stimulation.

Cardiac opioid receptors functionally and physically cross-talk with beta-adrenergic receptors via multiple hierarchical mechanisms, including heterodimerization of these receptors, counterbalance of functional opposing G protein signaling, and interface at downstream signaling events [35]. Moreover, opioid receptors such as KOR have been described to form heterodimers with the apelin receptors (APJ) and the AngII/AT1R system which are known regulators of the cardiovascular system [36–38]. The heterodimerization of APJ and ATR1 reduces the physiological effects of angII, and heterodimerization of APJ and KORs modulates cardiac contractility and intensifies the blood pressure-lowering effect of apelin. Therefore, the interaction between the apelin/APJ, opioid/OPRs, and ang II/ATR1 systems and the heterodimerization of their receptors during volume overload conditions, under which apelin levels are reduced, may contribute to the cardiovascular regulation in ACF-induced HF [36–38].

Liang et al. demonstrated already in 1987 that an upregulated opiate system may accompany CHF-induced sympathetic activation [39]. Effects of opioid receptor inhibition may, thus, be mediated via compensatory stimulation of the sympathetic nervous system or—more likely—by a reduced inhibition of the sympathetic nervous system. In the latter one, it is very intriguing that the cardiac actions of beta-adrenergic receptor (beta-AR) stimulation are attenuated by activation of the opioid receptor [40]. However, both states of increased sympathetic activity would hypothetically amplify the so-called vicious circle in cardiac remodeling resulting in increased workload reflected by increased BNP concentrations. We were able to demonstrate a significant reduction of the elevated rBNP-45 and angiotensin-2 levels in ACF rats treated with naltrexone. The RAAS, as part of the neurohumoral activation in heart failure, is also known to play a key role in fluid retention and cardiac remodeling [41]. The reduction in angiotensin-2 plasma levels might reflect an attenutated sympathetic activation in volume-overloaded rats with continuous naltrexone treatment. Sympathetic activation is known to induce inflammatory and apoptotic processes and is associated with adverse cardiac events [42, 43]. Inhibition of the RAAS significantly reduces cardiac fibrosis [44]. These findings might suggestthat reduced adrenergic activation might be associated with improved cardiac outcome.

Several limitations have to be considered. Firstly, these findings cannot directly be transfered to a clinical situation, since our experimental model does not represent the multimorbidity and causality of cardiac compromised patients. However, this animal model produces a very predictable state of nearly decompensated heart failure with a dilatative cardiomyopathy within 28 days. The Wistar rats showed overt signs of decompensation, e.g., ascites, strained breathing, decreased mobility, and sudden arrhythmia. Intriguingly, organ/body weight indices of the liver and kidney were not significantly altered in ACF rats which may be due to the large AV fistula itself that affects organ perfusion preventing it from increasing organ weight. Nonetheless, both organs showed overt histopathological changes in the liver [45] and kidney [46], as has been previously published. Secondly, this study was conducted to assess the indirect adverse opioidergic effects in cardiac volume overload. Therefore, the cardiac opioid system was blocked with an opioid receptor antagonist in a proof-of-concept design. Application of mu-, kappa-, and delta-opioid agonist would have been of benefit, but there already exists clinical data strongly supporting a negative influence of mu-opioid agonist [31]. In addition, naltrexone and its major metabolite, 6-β-natrexol, are known to mainly bind to mu-opioid receptors and to a lesser extent to kappa- and delta-opioid receptors (affinity (Ki) mu 1.0 nM, kappa 3.9 nM, delta 149 nM [47, 48]. We have not included a control group treated with naltrexone; however, naltrexone is known to lack any intrinsic activity at opioid receptors [49, 50]. The inhibition of the cardiac opioid system might be accompanied by centrally mediated effects. Thus, in future studies, effects of opioid receptor subtype selective agonists and antagonists with/without the ability to cross the blood brain barrier on cardiac function have to be investigated in an experimental setting. Thirdly, opioids can profoundly affect the respiratory system. The opioidergic effects on the respiratory system could likely be another significant consideration to explain the alteration of cardiac function. However, within the CRUSADE study, it has been demonstrated that acute and unique morphine administration during myocardial infarction resulted in an increased incidence of heart failure thereafter [31]. Unfortunately, we did not measure the breathing rate in all rats. But we can state that from a clinical point of view these rats appeared clinically inapparent during hemodynamic measurements. In this context, all measurements were performed under tiletamine/zolazepam anesthesia as this combination has to be found to elicit the least hemodynamic effect [18]. Thus, different anesthetic regimens might affect our findings. Our findings also have to be confirmed in other experimental models, e.g., myocardial infarction or pressure overload. Finally, the study was only conducted in male Wistar rats. Female Wistar rats are prone to a more difficult ACF induction, and standardization has not been established yet. Therefore, a comprehensive analysis of this nature amongst female Wistar rats has yet to be conducted.

In conclusion, the results of this experimental study give first-hand evidence of a cardiodepressant effect of the intrinsic cardiac opioid system during chronic volume overload. Thus, future studies have to address clinical opioid effects in perioperative patients with cardiovascular diseases.

## Data Availability

Not applicable.
